# Predictive Modeling Using Six-Month Performance Assessments to Forecast Long-Term Cognitive and Verbal Development in Pre-lingual Deaf Children With Cochlear Implants

**DOI:** 10.7759/cureus.78807

**Published:** 2025-02-10

**Authors:** Richi Sinha, Mani Mala, Rakesh K Singh

**Affiliations:** 1 Otolaryngology - Head and Neck Surgery, Indira Gandhi Institute of Medical Sciences, Patna, IND

**Keywords:** auditory outcomes, cochlear implants, longitudinal analysis, predictive modeling, speech intelligibility

## Abstract

Objective

This study aims to develop predictive models for speech outcomes at 6, 12, and 24 months post-cochlear implantation in pre-lingual deaf children. Using longitudinal Category of Auditory Performance (CAP), Speech Intelligibility Rating (SIR), and Parents' Evaluation of Aural/Oral Performance of Children (PEACH) scores, it seeks to forecast cognitive and verbal development. The study addresses the gap in correlating auditory performance with cognitive milestones by integrating longitudinal auditory data with cognitive and verbal benchmarks to identify predictive relationships.

Method

In this retrospective study, auditory performance data from hospital records of 157 post-cochlear implant children were analyzed using mixed-effects models, repeated measures ANOVA, and Tukey's HSD (honestly significant difference) post-hoc tests. The predictive value of outcomes at 6, 12, and 24 months was evaluated, focusing on temporal improvements and the interplay of demographic and procedural variables.

Results

The children had a mean implantation age of 3.7 years and a median switch-on time of 29 days; 58% were male. Their auditory and speech performance demonstrated significant improvement over time, with CAP scores increasing from 1.56 at 6 months to 4.55 at 24 months, SIR scores improving from 1.03 to 2.04, and PEACH scores rising from 17.91 to 38.14 (p < 0.0001 for all). Predictive modeling revealed that early improvements at 6 and 12 months were strong indicators of speech and cognitive outcomes at 24 months. The findings highlight significant predictive relationships, demonstrating that early auditory performance assessments correlate with later cognitive and verbal competencies.

Conclusion

This study demonstrates that early auditory outcomes at 6 and 12 months can reliably predict long-term developmental trajectories following cochlear implantation. It establishes a framework for integrating predictive analytics into pediatric audiology, enhancing speech and cognitive outcomes for pre-lingual deaf children.

## Introduction

The landscape of pediatric audiology continues to evolve, driven by advancements in technology such as cochlear implants and improved early intervention strategies for children with hearing impairments. Recent studies have highlighted the critical importance of early auditory exposure and its impact on language acquisition and cognitive development in pre-lingual deaf children, underscoring the relationships between auditory performance and cognitive outcomes [1,2]. Nevertheless, despite the proliferation of cochlear implants over the past few decades, substantial variability remains in the cognitive and verbal development trajectories of these children post-implantation, raising significant concerns regarding the effectiveness of current predictive models and assessments [3-5]. The primary research problem in this domain is the under-exploration of longitudinal relationships between auditory performance, particularly as assessed by the Six-Month Auditory Performance Scale, and cognitive as well as verbal milestones in this unique population. Although previous studies highlight the overall success of cochlear implants and factors like age at implantation and rehabilitation practices, the existing literature lacks robust predictive frameworks that can accurately correlate 6- and 12-month auditory performance metrics with later developmental outcomes, thereby limiting clinicians' ability to tailor interventions effectively to individual needs [6].

Research has shown that cochlear implants significantly improve auditory performance, with documented gains in CAP (Category of Auditory Performance), SIR (Speech Intelligibility Rating), and PEACH (Parents' Evaluation of Aural/Oral Performance of Children) scores. However, these studies tend to emphasize aggregate improvements over time rather than examining how early milestones influence later outcomes [7,8]. The objectives of the current study revolve around developing a comprehensive predictive model that utilizes longitudinal data to evaluate the relationships between early auditory performance and subsequent cognitive and verbal abilities, with a particular emphasis on using the Six-Month Auditory Performance Scale as a baseline assessment tool. By systematically analyzing these connections, the research seeks to establish reliable predictive indicators that can be integrated into clinical practice, thereby enhancing early intervention strategies.

Analyzing the predictive value of auditory outcomes at 6 and 12 months post-implantation can provide actionable insights for clinical practice. Specifically, gains in CAP, SIR, and PEACH scores during the early post-implantation period are expected to correlate with long-term success. Improved predictive modeling holds the potential for refining and personalizing treatment plans, ensuring that interventions are grounded in evidence-based practices that optimize developmental outcomes. Furthermore, such advancements can inform policy-making and resource allocation within pediatric audiology, driving initiatives that prioritize early and continuous auditory performance assessments in this vulnerable population. Ultimately, the findings of this research could foster a paradigm shift in how auditory performance data is utilized to enhance the quality of life for children who are deaf, enabling them to achieve their full communicative and cognitive potential.

## Materials and methods

Study design and setting

This retrospective, unicentric, longitudinal study was conducted in the Otorhinolaryngology Department of Indira Gandhi Institute of Medical Sciences from September 2017 to December 2023. Ethical approval was obtained from the Institutional Ethics Committee, Indira Gandhi Institute of Medical Sciences, Patna, India (approval number: 1376/IEC/IGIMS/2024, dated 20/03/2024).

Study participants

We reviewed the hospital records of all children under 14 years of age diagnosed with bilateral severe-to-profound sensorineural hearing loss who underwent cochlear implantation at our institute. Inclusion criteria were limited to prelingual deaf children who underwent unilateral cochlear implant surgery and completed at least 24 months of auditory-verbal therapy. Exclusion criteria included children with post-lingual deafness, multiple disabilities, poor compliance, less than 24 months of adherence to the prescribed auditory-verbal therapy protocol, or incomplete records. Of the 220 children who underwent cochlear implant surgery, 157 met the criteria and were included in the study.

Sample size

The sample size was determined using a repeated measures ANOVA design to ensure adequate statistical power for detecting medium-sized effects (Cohen's d = 0.25) across three measurement points. Calculations were performed using XLSTAT, incorporating an average intra-class correlation of 0.824 for CAP score (based on a pilot study of 30 patients) to account for the high correlation among repeated measures within individuals. To achieve a power of 85% with an alpha level of 0.05, the analysis indicated that 157 participants were required. This sample size provides sufficient power to detect clinically significant changes in auditory performance, ensuring robust findings over the study period.

Data collection

Data were collected from existing medical records, including demographic and medical history, comprehensive otolaryngological examinations, and audiological assessments such as tympanometry, pure-tone audiometry, brainstem evoked response audiometry, otoacoustic emissions, and auditory steady-state response. Operative records confirmed that unilateral cochlear implantation was performed by the same experienced surgeon, following a consistent surgical protocol. All participants received Cochlear™ Nucleus® CI422 cochlear implants with slim straight electrodes (Cochlear Ltd., Australia). Post-implantation, after device activation with the Nucleus 5 Sound Processor (CP810) supported by the Cochlear™ Nucleus® CR110 Remote Control (Cochlear Ltd., Australia), all children underwent standardized auditory-verbal therapy (AVT). Therapy was conducted by a single certified AVT specialist at the center, focusing on auditory skill development and spoken language acquisition, with sessions tailored to individual progress based on auditory-verbal principles. Auditory and verbal outcomes were assessed at 6, 12, and 24 months of AVT using revised CAP, SIR, and PEACH scores, collected during routine follow-up visits.

Statistical analysis

All statistical analyses were conducted using Microsoft Excel (v16.89, Microsoft Corporation, Redmond, Washington) and XLSTAT (v2024.4.0.1424, ADDINSOFT, Paris, France). The study examined the predictive value of early speech and language scores at 6 and 12 months to determine long-term outcomes. Summary statistics were calculated for all continuous variables, including means, standard deviations, medians, and interquartile ranges. Visual assessments were performed using violin plots. The Shapiro-Wilk test confirmed the non-normal distribution of variables, leading to the use of Spearman’s rank correlation for analysis. A mixed-effects model accounted for intra-individual differences over time and determined the effect of confounding factors such as age at implantation, gender, and duration of device switch-on. Repeated measures ANOVA was used to confirm findings. Diagnostic checks were performed to assess the adequacy of the model and ensure that underlying assumptions were met. Mauchly’s Test of Sphericity was applied to test for equality of variances among all combinations of related groups. Greenhouse-Geisser corrections were applied in cases of sphericity violations. The normality of residuals was inspected through Q-Q plots. Levene’s test assessed the homogeneity of variances across groups. The linearity of relationships was examined through scatter plots of residuals against predicted values. Model fit was verified by analyzing the overall F-test for significance and calculating the root mean square error (RMSE) to measure how well the model predictions matched the observed data. Post-hoc pairwise comparisons were conducted using Tukey’s HSD test following significant ANOVA results to identify specific differences between measurement occasions. The scores were divided into binary variables, good and poor, using mean values as the threshold. ROC analysis was utilized to establish predictive cutoffs for 6- and 12-month scores regarding 24-month outcomes.

## Results

**Figure 1:**
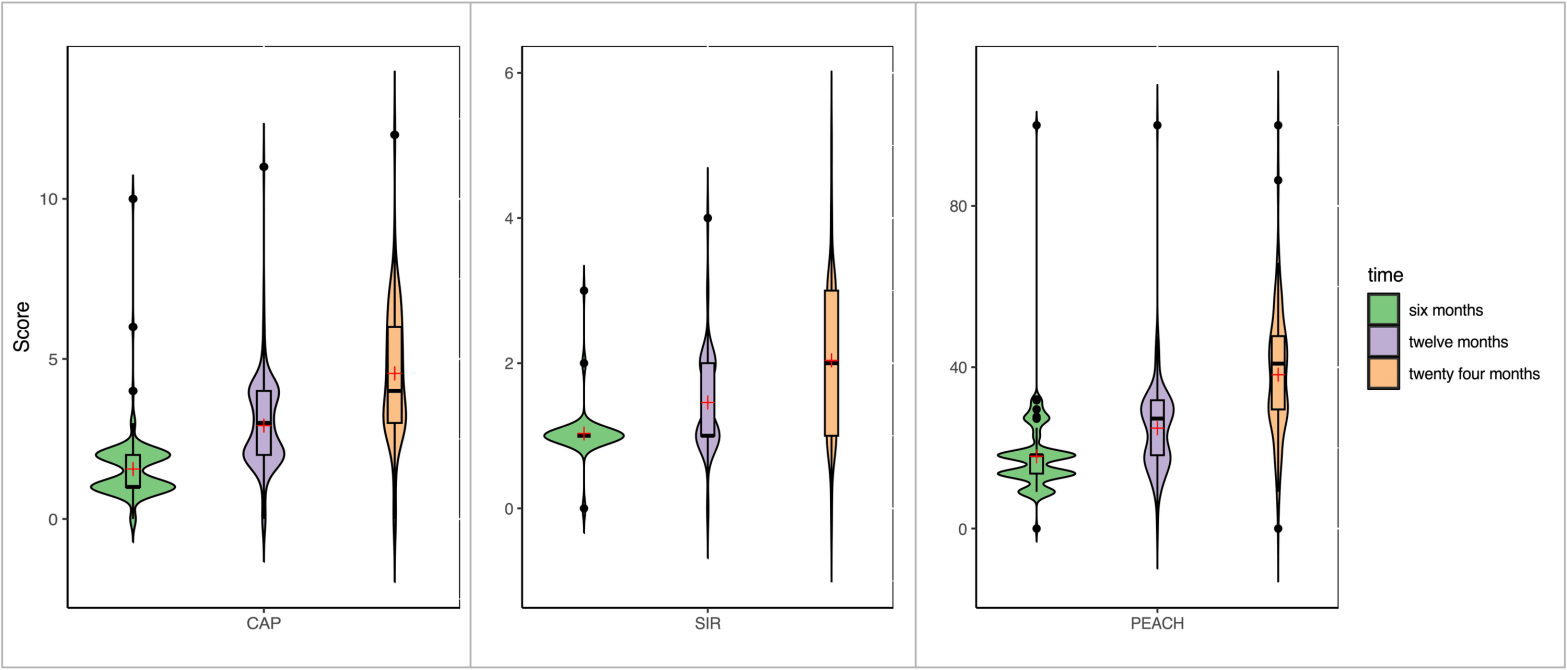
Violin plots showing the distribution of auditory and speech assessment scores (CAP, SIR, and PEACH) over time following cochlear implantation Each violin plot represents the score distribution at a specific time point, with the width indicating the density of observations. Inside each violin plot, a box plot highlights the interquartile range, with a black bar indicating the median score and a red plus sign denoting the mean score. CAP: Category of Auditory Performance, SIR: Speech Intelligibility Rating, PEACH: Parents' Evaluation of Aural/Oral Performance of Children.

This study assessed auditory outcomes in pre-lingual deaf children with cochlear implants, utilizing a longitudinal dataset to examine the impact of time post-implantation on revised CAP, SIR, and PEACH scores. It included 157 children with a mean age at implantation of 3.71 ± 2.08 years (median: 3 years; IQR: 1.75), ranging from 9 months to 14 years. The average time to switch on was 40.5 ± 48.1 days (median: 29 days; IQR: 13), with 57.96% being male (Table 1). Their mean scores over time are summarized in Table 2. Violin plot data indicate increasing variability and median scores across all metrics as time progresses, with CAP and SIR showing gradual improvement, while PEACH demonstrates significant enhancement in real-world auditory performance. The broadening distributions suggest individual differences in the practical application of auditory skills in daily environments (Figure 1). To analyze the temporal dynamics of auditory development, we employed two analytical approaches: repeated measures ANOVA and a mixed-effects model.

**Table 1:** Demographic characteristics of study participants IQR: interquartile range.

Variables	N	Percentage	Mean ± SD	Minimum	Maximum	Median (IQR)
Age at implant (in years)	157	-	3.71 ± 2.08	0.75	14.00	3 (1.75)
Time to switch on (in days)	157	-	40.50 ± 48.10	12.00	395.00	29 (13)
Sex (male)	91	57.96	-	-	-	-
Sex (female)	66	42.04	-	-	-	-

**Table 2:** Summary of statistical analyses for auditory outcomes in pre-lingual deaf children with cochlear implants CAP: Category of Auditory Performance, SIR: Speech Intelligibility Rating, PEACH: Parents' Evaluation of Aural/Oral Performance of Children, SD: standard deviation, RMSE: root mean square error, Tukey HSD: Tukey honestly significant difference.

Metric	Time Points	CAP Scores	SIR Scores	PEACH Scores
Mean scores ± SD	6 months	1.56 ± 0.99	1.03 ± 0.34	17.91 ± 9.20
	12 months	2.92 ± 1.36	1.46 ± 0.70	24.89 ± 10.63
	24 months	4.55 ± 2.00	2.04 ± 1.03	38.14 ± 15.26
Regression coefficients	Intercept	1.561	1.032	17.910
	6 months	Ref.	Ref.	Ref.
	12 months	1.357	0.427	6.981
	24 months	2.987	1.006	20.227
Standard error	12 months	0.171	0.085	1.356
	24 months	0.171	0.085	1.356
P-value	12 months	< 0.0001	< 0.0001	< 0.0001
	24 months	< 0.0001	< 0.0001	< 0.0001
R²	-	0.394	0.233	0.329
RMSE	-	1.519	0.750	12.019
Tukey HSD results	12 vs 6 months	1.357 (p<0.0001)	0.427 (p<0.0001)	6.981 (p<0.0001)
	24 vs 12 months	1.631 (p<0.0001)	0.580 (p<0.0001)	13.246 (p<0.0001)
	24 vs 6 months	2.987 (p<0.0001)	1.006 (p<0.0001)	20.227 (p<0.0001)

Repeated measures ANOVA

**Figure 2:**
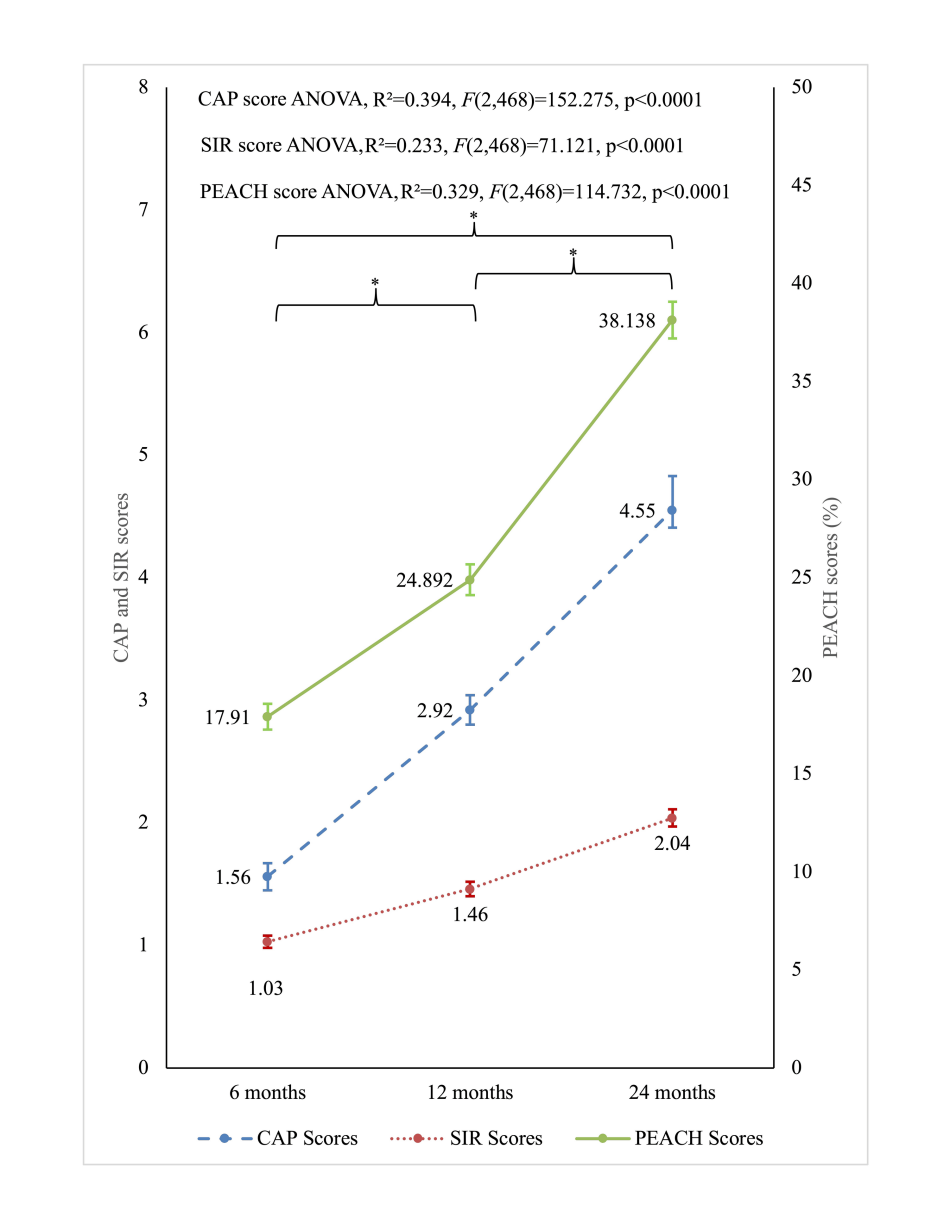
Line graph displaying the trajectory of CAP, SIR, and PEACH scores over 6, 12, and 24 months Error bars denote 95% confidence intervals. Significant improvements are marked with an asterisk (p < 0.0001) between each time point, and ANOVA statistics demonstrate substantial model efficacy for the scores. CAP: Category of Auditory Performance, SIR: Speech Intelligibility Rating, PEACH: Parents' Evaluation of Aural/Oral Performance of Children.

The analysis focused on the within-subjects effects of time on scores and demonstrated significant improvement in the mean scores over time (p < 0.0001 for all). The results are summarized in Table 2. Since Mauchly's test indicated that the assumption of sphericity had been violated, χ²(2) = 2653.30, p < .0001, the degrees of freedom were corrected using Greenhouse-Geisser estimates of sphericity (ε = 0.503). As illustrated in Figure 2, all three auditory and speech performance measures exhibited substantial improvements from 6 to 24 months post-implantation, with the greatest influence of AVT duration observed with CAP scores, suggesting that extended therapy durations correlate with more substantial improvements in auditory capabilities over time.

Mixed-effects model analysis

The mixed-effects model incorporated time as a fixed effect and subject variability as a random effect to account for intra-individual differences. Age at implantation generally had a negative impact on initial CAP and PEACH scores, suggesting that younger implantation ages may be beneficial while having minimal effect on SIR scores. However, this association was statistically insignificant (p = 0.665, 0.774, and 0.266 for CAP, SIR, and PEACH scores, respectively). The time to switch on the device showed a slight negative impact across all scores, indicating that faster activation may be advantageous. However, this association was also statistically insignificant (p = 0.287, 0.253, and 0.484 for CAP, SIR, and PEACH scores, respectively). Gender analysis revealed no significant differences in scores between males and females, suggesting that gender does not impact the overall effectiveness of cochlear implants in enhancing auditory performance in the study subjects. The progression from 6 to 12 and 24 months showed marked increases across all scores (all p < 0.0001), as described in Table 3, underscoring the substantial benefits of cochlear implants over time. The random effects parameter indicated substantial inter-individual variability (variance = 1.175, p < 0.0001), highlighting the diverse trajectories of auditory recovery among children. This insight is pivotal for clinicians aiming to optimize intervention strategies based on patient-specific temporal dynamics rather than relying solely on static demographic factors.

**Table 3:** Summary of mixed-effects model results for CAP, SIR, and PEACH scores over 24 months *p < 0.0001. CAP: Category of Auditory Performance, SIR: Speech Intelligibility Rating, PEACH: Parents' Evaluation of Aural/Oral Performance of Children, SE: standard error.

Parameter	CAP Score	SIR Score	PEACH Score
Intercept (SE)	1.834 (0.281)	1.057 (0.127)	21.119 (2.133)
Age at implant (SE)	-0.024 (0.054)	0.007 (0.025)	-0.483 (0.434)
Time to switch on (SE)	-0.002 (0.002)	-0.001 (0.001)	-0.013 (0.019)
Sex - female (SE)	-0.205 (0.227)	-0.004 (0.106)	-2.103 (1.818)
6 months (SE)	Ref.	Ref.	Ref.
12 months (SE)	1.357 (0.171)*	0.427 (0.052)*	6.981 (0.616)*
24 months (SE)	2.987 (0.171)*	1.006 (0.052)*	20.227 (0.616)*

Tukey HSD post-hoc analysis

The Tukey HSD test revealed statistically significant differences between all pairs of time points, with maximum progress between 6 and 24 months, underlining substantial gains in auditory perception over time (Table 2).

Correlation analysis

**Figure 3:**
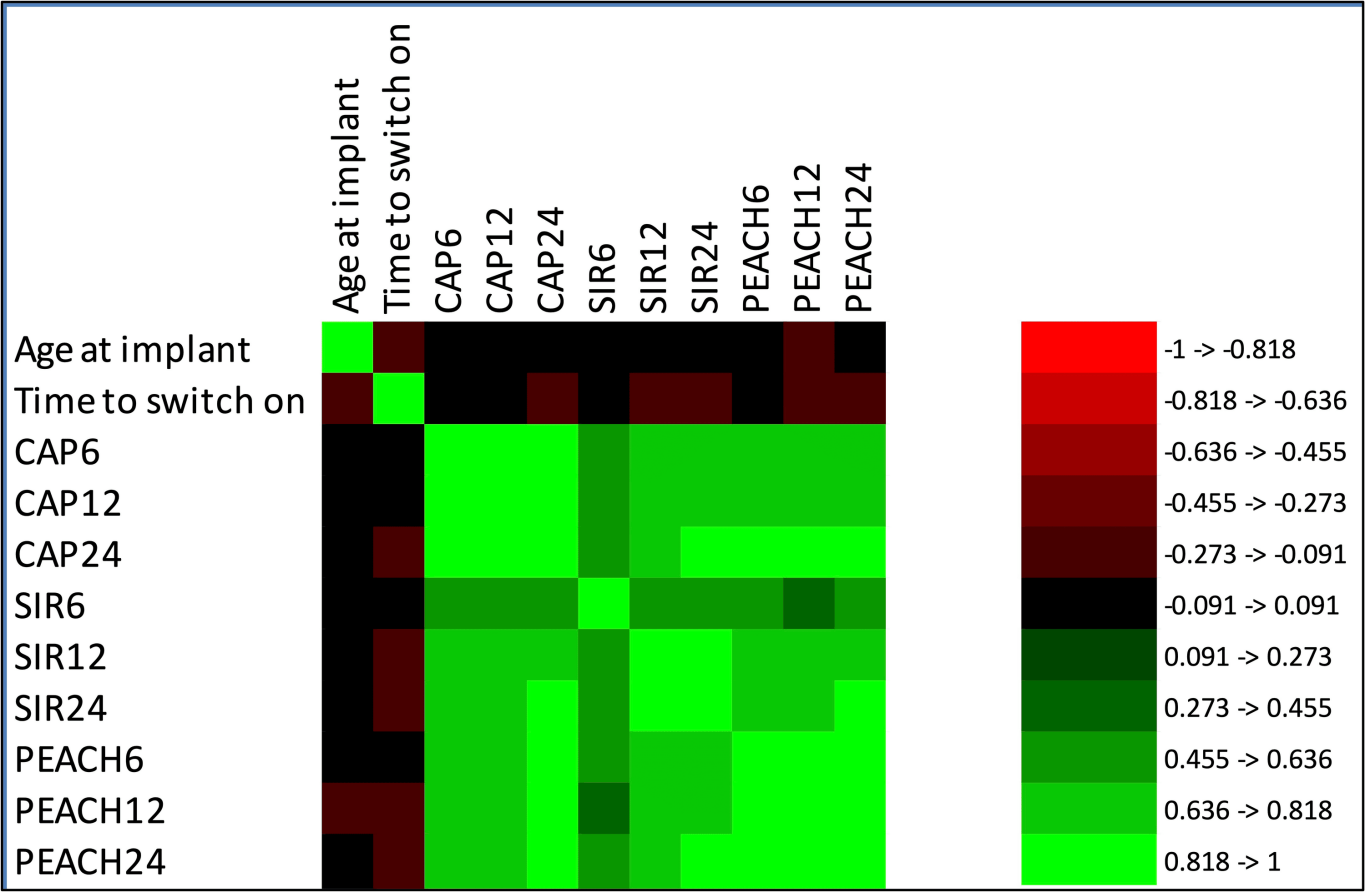
Heatmap of correlation coefficients among demographic, procedural variables, and auditory performance scores at 6, 12, and 24 months post-implantation Red indicates a strong negative correlation, black represents no correlation, and green shows a strong positive correlation. The scale on the right explains the color coding relative to correlation strength, ranging from -1 (perfect negative correlation) to +1 (perfect positive correlation). CAP: Category of Auditory Performance, SIR: Speech Intelligibility Rating, PEACH: Parents' Evaluation of Aural/Oral Performance of Children.

In addition to the primary statistical models, a comprehensive correlation analysis was conducted to evaluate the relationships between demographic and procedural variables with auditory and speech outcomes (Figure 3). This analysis played a crucial role in understanding the impact of non-temporal factors on the effectiveness of cochlear implants. Our analysis largely revealed no significant correlations between demographic covariates, such as age at implantation, and auditory performance scores (CAP, SIR, PEACH) across all time points. Similarly, procedural variables, like the time to switch on, showed no consistent influence on CAP and PEACH scores, indicating that these variables do not typically affect cochlear implantation outcomes. A notable exception was observed in the relationship between time to switch on and SIR scores at 24 months. A statistically significant but modest negative correlation was identified (coefficient = -0.162, p = 0.043), suggesting that longer delays in activating the cochlear implant are associated with slightly lower speech intelligibility outcomes at this later stage. This finding emphasizes the importance of timely device activation to enhance speech intelligibility outcomes.

Furthermore, our analysis confirmed strong positive correlations among the different scores (CAP, SIR, PEACH) across the three time points. These correlations indicate that improvements in one domain of auditory or speech performance are generally accompanied by improvements in others, reflecting the integrated nature of auditory development facilitated by cochlear implants. The absence of significant correlations with most covariates suggests that the primary determinant of success in cochlear implantation, in terms of auditory and speech outcomes, is less dependent on individual demographic or procedural variations and more on the inherent effectiveness (like due to genetic factors) of cochlear implant technology and rehabilitation processes.

ROC analysis

The mean values for CAP, SIR, and PEACH scores at 6 and 12 months were used as thresholds to define "good" and "poor" outcome categories for predicting scores at 24 months (Table 4).

**Table 4:** Predictive performance of early scores in forecasting long-term outcomes Mean values for CAP, SIR, and PEACH scores at 6 and 12 months were used as thresholds to define 'good' and 'poor' outcome categories to predict scores at 24 months. * p-value = 0.002, **p-value < 0.001. AUC: area under the curve, CAP: Category of Auditory Performance, SIR: Speech Intelligibility Rating, PEACH: Parents' Evaluation of Aural/Oral Performance of Children.

Metrics	CAP (6 Months)	CAP (12 Months)	SIR (6 Months)	SIR (12 Months)	PEACH (6 Months)	PEACH (12 Months)
Sensitivity (%)	85.3	93.3	14.5	98.2	91.5	93.9
Specificity (%)	86.6	84.9	100	88.2	88.0	82.7
Positive predictive value (%)	85.3	85.4	100	81.8	89.3	85.6
Negative predictive value (%)	86.6	93.3	68.5	98.9	90.4	92.5
Accuracy (%)	86.0	89.2	70.1	91.7	89.8	88.5
AUC	0.870**	0.893**	0.573*	0.932**	0.897**	0.883**

CAP Scores

An AUC of 0.870 indicates good predictive ability. Children scoring two or more on CAP at 6 months are likely to have 'good' outcomes at 24 months, suggesting that early CAP scores are a reliable indicator of long-term auditory development.

SIR Scores

An AUC of 0.573 indicates poor predictive ability at 6 months, suggesting that early SIR scores do not reliably predict outcomes at 24 months. However, a higher AUC of 0.932 at 12 months indicates a stronger predictive value over time, suggesting that SIR scores become more predictive over time.

PEACH Scores

An AUC of 0.897 indicates good predictive ability. Higher PEACH scores at 6 months (> 17.89) significantly predict better verbal and cognitive development at 24 months, reinforcing the importance of early auditory and verbal experiences.

**Figure 4:**
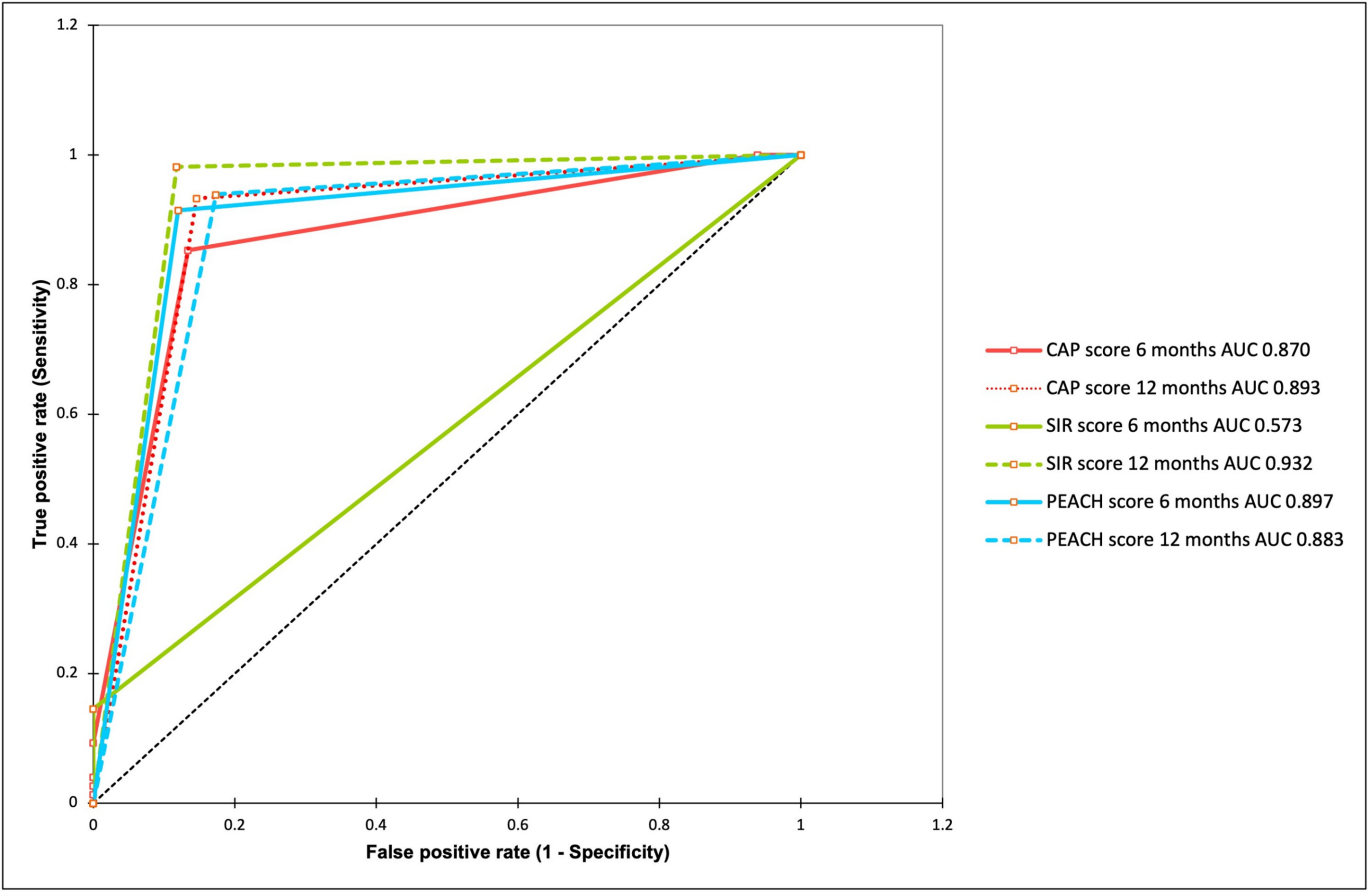
ROC curves for CAP, SIR, and PEACH scores at 6 and 12 months This figure illustrates the predictive accuracies of early CAP, SIR, and PEACH scores for long-term outcomes at 24 months post-cochlear implantation, measured by the area under the curve (AUC). CAP and PEACH scores at 6 months demonstrate robust predictive capabilities (AUC > 0.85), while SIR scores at 6 months show limited predictive utility. CAP: Category of Auditory Performance, SIR: Speech Intelligibility Rating, PEACH: Parents' Evaluation of Aural/Oral Performance of Children.

With their high AUC values, CAP and PEACH scores at 6 and 12 months serve as strong tools for early prediction of long-term outcomes in cochlear implant recipients (Figure 4).

## Discussion

The findings of this study validate the significant and progressive impact of cochlear implants on auditory and speech outcomes in prelingual deaf children, with improvements demonstrated over 6, 12, and 24 months post-implantation. By integrating results with prior research and current data, this study offers a comprehensive perspective on auditory development facilitated by cochlear implants and provides actionable insights for clinical practice.

This study’s results align with previous literature, emphasizing the transformative potential of cochlear implants. Blamey et al. (2013) highlighted the critical importance of early auditory exposure in fostering speech perception and language acquisition [9]. Our findings corroborate this, showing significant improvements in CAP scores, from a baseline of 1.56 ± 0.99 at 6 months to 4.55 ± 2.00 at 24 months (p < 0.0001), underscoring rapid adaptation to auditory input during early post-implantation stages. Similarly, Connor et al. (2006) and Kral et al. (2016) emphasized heightened neuroplasticity in young children [10,11], which is reflected in the steady rise in SIR scores, from 1.03 ± 0.34 at 6 months to 2.04 ± 1.03 at 24 months (p < 0.0001) in our study population. These findings reaffirm the importance of timely intervention and structured rehabilitation to optimize outcomes.

A unique aspect of this study is the integration of PEACH scores, which evaluate real-world auditory functionality. Previous research has primarily focused on controlled clinical metrics, often overlooking the everyday implications of cochlear implant use. Our findings show a significant increase in PEACH scores, from 17.91 ± 9.20 at 6 months to 38.14 ± 15.26 at 24 months (p < 0.0001), demonstrating how auditory gains translate into enhanced social engagement and communication in real-world settings. This practical dimension highlights the broader impact of cochlear implants on a child’s quality of life, bridging the gap between clinical assessments and lived experiences.

Time emerged as the most critical determinant of outcomes. Our research demonstrated significant differences across time intervals in CAP scores, with Δ = 1.357 between 6 and 12 months and Δ = 2.987 between 6 and 24 months. These findings emphasize the necessity of sustained auditory exposure and therapy, particularly during the first two years post-implantation. The 6- to 12-month period appears to be a critical phase for consolidating early gains, aligning with findings from Abdullah et al. (2020). Their study observed stabilization in auditory performance during this timeframe. They evaluated CAP and SIR scores at two points within six months post-implantation in children under four years of age. The results demonstrated significant improvements in CAP and SIR scores during the first and second assessments (p = 0.040 and p = 0.034, respectively). Additionally, they noted that prolonged cochlear implant use significantly enhanced SIR scores (p = 0.011) [12].

While earlier studies often prioritized demographic factors, such as age at implantation, as key predictors of success [13,14], this study found no significant influence of age or gender on CAP and PEACH scores. This contradicts research by Noblitt et al., which suggested that multiple barriers, including time and individual engagement, low socioeconomic status, parental education, and access to rehabilitation services, have greater impacts on speech and language development [15]. However, inter-individual variability, as evidenced by the random effects variance of 1.175 (p < 0.0001) in CAP scores, highlights the diverse developmental trajectories among children. Therefore, personalized rehabilitation strategies, tailored to each child’s unique needs and learning capacities, are essential for optimizing outcomes.

The relationship between delayed activation of the device and slightly lower SIR scores at 24 months (coefficient = -0.162, p = 0.043) further emphasizes the importance of timely activation. This finding aligns with Emin, who demonstrated that implantation delays can hinder speech intelligibility development [16]. However, our analysis found no consistent impact of other procedural variables, such as time to switch on CAP and PEACH scores, suggesting that ongoing therapy and auditory adaptation play a more decisive role in long-term success.

A notable strength of this study is the inclusion of multiple metrics (CAP, SIR, and PEACH scores) to provide a multidimensional view of auditory development. Consistent with the work of Liu et al. and Radhakrishnan et al., our findings revealed strong positive correlations among these measures, indicating that progress in one domain often complements gains in others [17,18]. This interconnectedness highlights the need for comprehensive rehabilitation strategies that simultaneously address auditory perception, speech intelligibility, and real-world functionality.

The implications of these findings are significant for clinical practice. The 6-month evaluation provides a critical opportunity to identify children who may require intensified or alternative interventions. At the same time, the 12-month milestone serves as a key checkpoint to assess progress and refine therapy strategies. By 24 months, outcomes offer a reliable benchmark for long-term success, enabling clinicians to make informed decisions about continuing or modifying rehabilitation efforts. Including real-world functionality measures, such as the PEACH score, further underscores the importance of interventions that lead to meaningful improvements in daily life.

Despite these strengths, certain limitations must be acknowledged. The exclusion of socioeconomic factors, parental involvement, and educational access limits the generalizability of findings to more diverse populations. Previous research, including studies by Guo et al., has shown that contextual variables significantly influence auditory rehabilitation outcomes, particularly in resource-limited settings [8]. Additionally, focusing on three time points may have overlooked finer temporal patterns in auditory development. More frequent assessments could reveal nuanced trajectories and fluctuations, particularly during periods of rapid adaptation.

Future research should address the gaps identified in this study. Incorporating socioeconomic, environmental, and cognitive variables would enhance the generalizability of findings and provide a more holistic understanding of cochlear implant success. Longitudinal studies with more frequent assessments and diverse populations could offer deeper insights into the temporal dynamics of auditory development. At the same time, qualitative measures, such as family interviews and psychosocial evaluations, would enrich our understanding of the broader impacts of cochlear implantation.

## Conclusions

This study underscores the transformative impact of cochlear implants in unlocking auditory and speech potential for pre-lingual deaf children. Over two years, significant gains were observed across CAP, SIR, and PEACH scores, with time emerging as the most decisive factor driving these improvements. Timely activation and individualized care were instrumental in optimizing outcomes, while demographic factors such as age and gender played a minimal role. The variability in individual trajectories highlights the importance of tailoring strategies to each child’s unique needs. The inclusion of real-world performance metrics further emphasizes the profound impact of cochlear implants on daily life, bridging clinical success with tangible improvements in social and functional engagement. These findings serve as a valuable guide for clinicians, families, and policymakers, helping to refine care strategies and maximize the transformative potential of cochlear implants. By empowering children to achieve richer auditory and communicative experiences, cochlear implants significantly enhance their quality of life.
